# Encountering the accessory polar renal artery during retroperitoneal lymphadenectomy

**DOI:** 10.1002/ccr3.3493

**Published:** 2020-12-12

**Authors:** Ceyda Karadağ, Ozer Birge, Mehmet Sait Bakir, Selen Doğan, Hasan Aykut Tuncer, Tayup Şimşek

**Affiliations:** ^1^ Department of Gynecological Oncology Akdeniz University Faculty of Medicine Antalya Turkey

**Keywords:** accessory artery, accessory polar renal artery, polar artery, vascular anomalies

## Abstract

Because of the accessory polar renal artery (APRA) is functional, it is extremely important to be careful with vascular injuries, to prevent ischemic damage and not to cause kidney failure complications.

## Graphical abstract

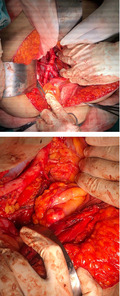

## 1 INTRODUCTION

The kidneys are normally blooded from renal artery that anatomically originates from the abdominal aorta. However, there are accessory polar artery variations. Incidence varies ranges from 11.3% to 59.5% depending on ethnicity. We also wanted to show two different cases of accessory polar renal artery.

The kidneys are normally blooded from renal artery that anatomically originates from the abdominal aorta. However, there are accessory polar artery variations.^1^, ^2^ Incidence varies ranges from 11.3% to 59.5% depending on ethnicity.^3^, ^4^ In a cadaver dissection study, the incidence of accessory polar renal artery in women and men was not different; in the same study, it was found that the variation of polar artery was more often in the right kidney than in the left kidney.^5^ We also wanted to show two different cases of accessory polar renal artery (APRA) that we operated for endometrial cancer and detected during paraaortic lymph node dissection.

## 2 CASE REPORT

A 53‐year‐old woman, body mass index of 27.2, gravida 1, parity 1, and had a previous cesarean section. Patient applied to our clinic for postmenopausal bleeding. Endometrial sampling result was endometrioid type endometrium cancer, histologic grade 2, and nuclear grade 3.

**FIGURE 1:**
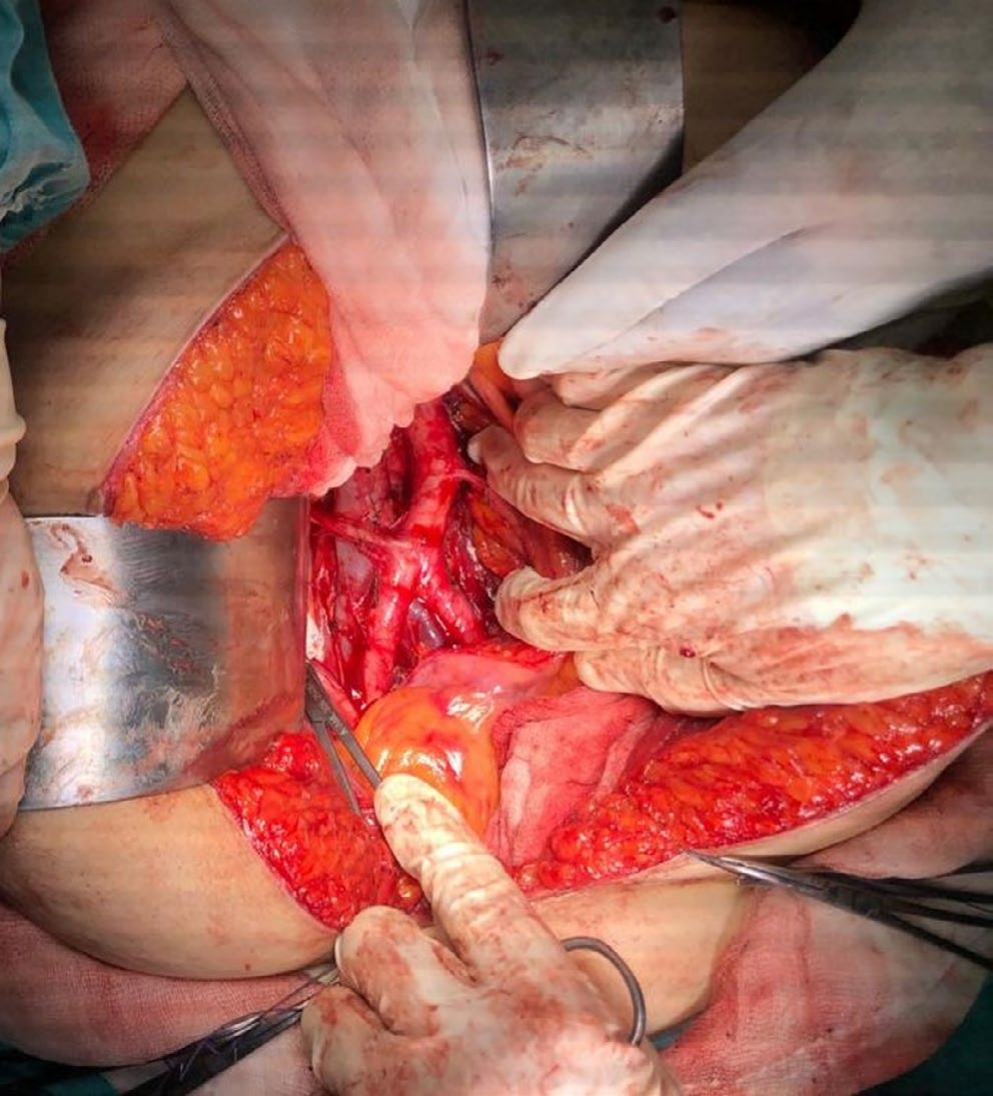
Accessory polar renal artery was detected from bifurcation of abdominal aorta, beginning of the right common iliac artery to right kidney

Preoperative MR imaging, it was reported that myometrial invasion was more than 1/2 and there was a 3.5 cm tumor in the uterine cavity. The patient underwent laparotomy, hysterectomy, bilateral salpingo‐ooferectomy, infracolic omentectomy, and bilateral pelvic‐paraaortic lymph node dissection. During the paraaortic lymph node dissection, APRA was detected from bifurcation of abdominal aorta, beginning of the right common iliac artery to right kidney (Figure 1). The operation is completed without any vascular complications.

**FIGURE 2:**
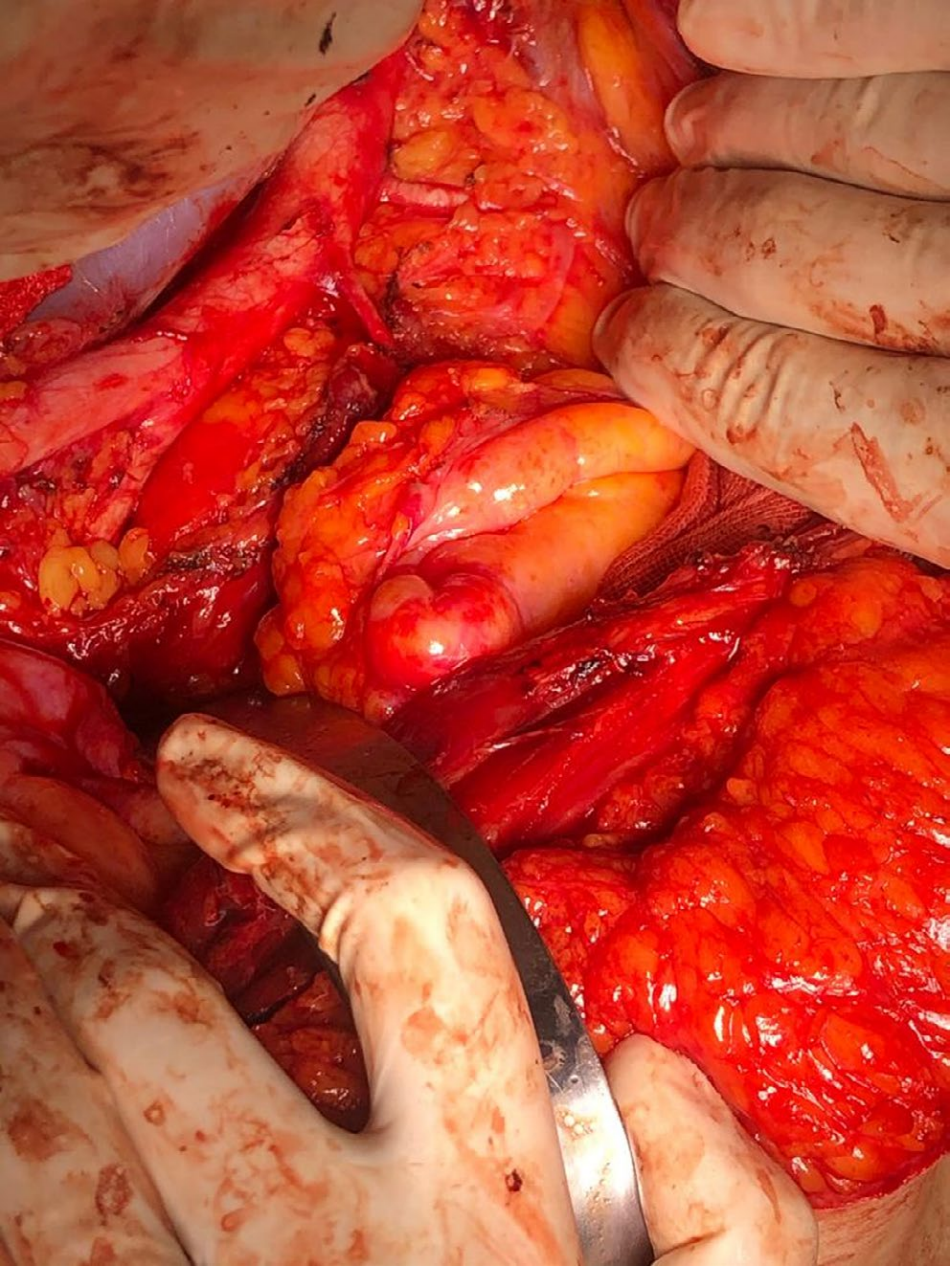
Accessory polar renal artery was observed approximately 1.5 cm above the inferior mesenteric artery

The other case is a 59‐year‐old woman, body mass index of 30, gravida 1, parity 1, and she had not previous operation. She applied to the clinic for postmenopausal bleeding. Transvaginal ultrasonography showed a hematometra of 10 cm in the uterine cavity. Endometrial sampling result was endometrioid type endometrium cancer, histologic grade 1, nuclear grade 2. Preoperative MR imaging, it was reported that myometrial invasion was more than 1/2. The patient underwent laparotomy, hysterectomy, bilateral salpingo‐ooferectomy, infracolic omentectomy, and bilateral pelvic‐paraaortic lymph node dissection. During the paraaortic lymph node dissection, the left APRA was observed approximately 1.5 cm above the inferior mesenteric artery. The operation is completed without any complications (Figure 2).

## 3 DISCUSSION

Because of the APRA is functional, it is extremely important to be careful with vascular injuries, to prevent ischemic damage and not to cause kidney failure complications.^6^ In addition, cases of reno‐vascular hypertension due to polar artery injury have been reported in the literature.^7^ Any vascular anomalies were reported in the preoperative imaging tests performed in both cases. For this reason, it is very important that gynecologist oncologists surgical experience and know the retroperitoneal vascular anatomy well, because of the possibility of encountering vascular variations.

## CONFLICT OF INTEREST

None declared.

## AUTHOR CONTRIBUTIONS

CK, OB**:** Manuscript writing. MSB, HAT: Manuscript design. SD, T.Ş: Revision and supervision.

## ETHICAL APPROVAL

Informed consent was obtained from the patients. Our institution does not require ethical approval for case reports.

## Data Availability

All patient data are available.
